# Lethal multiple pterygium syndrome in a newborn, a case report

**DOI:** 10.1002/ccr3.7678

**Published:** 2023-07-12

**Authors:** Parvaneh Sadeghimoghadam, Saeedeh Shirdel, Sedigheh Hantoushzadeh, Zeinab Hashemi, Marjan Ghaemi

**Affiliations:** ^1^ Department of Pediatrics, Vali‐E‐Asr Hospital, Imam Complex Tehran University of Medical Sciences Tehran Iran; ^2^ Vali‐E‐Asr Reproductive Health Research Center Family Health Research Institute, Tehran University of Medical Sciences Tehran Iran

**Keywords:** congenital disorders, genetic counseling, genetic screening, multiple pterygium syndrome, musculoskeletal anomaly

## Abstract

Lethal multiple pterygium syndrome is a very rare genetic disorder. The manifestations of this condition include growth deficiency of the fetus, craniofacial anomalies, joint contracture, and skin webbing (pterygia). This disorder is fatal before birth or shortly after birth. We reported a case of lethal multiple pterygium syndrome with multiple anomalies including pterygia involving the axilla, bilateral antecubital fossa, and groin. Arthrogryposis involving multiple lower and upper extremities joints. Cleft palate, microstomia and limitation of mouth opening, webbed neck, asymmetric small and narrow chest, ambiguous genitalia, depressed and wide nasal bridge, antemongoloid slant, low‐set, malformed, and posteriorly rotated ears, pterygia, syndactyly and camptodactyly of hands and rocket bottom feet. LMPS is a congenital genetic disease with multiple anomalies that is fatal in the second and third trimesters of pregnancy or shortly after birth. With genetic testing and counseling, it can be prevented from recurring in subsequent pregnancies.

## Graphical abstract

A newborn with pterygium underwent advanced resuscitation in the operating room.

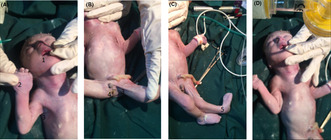

## 1 INTRODUCTION

Multiple pterygium syndrome (MPS) is a rare genetic disorder that is characterized by arthrogryposis (joint contractures), skin webs on the joints (pterygia), short stature, kyphoscoliosis, craniofacial anomalies and other anomalies of bones, joints, and limb.^1^, ^2^ MPS includes two major forms: lethal and nonlethal (also called Escobar syndrome). The lethal form causes death during infancy (in the second or third trimester) or shortly after birth.^3^, ^4^, ^5^ The main inheritance pattern of the disease is autosomal recessive) autosomal dominant and X‐linked have also been reported).^6^, ^7^, ^8^

This syndrome is generally diagnosed during the prenatal period by fetal sonography, which shows evidence of oligohydramnios, arthrogryposis, and reduced fetal movements.^5^ Due to the rare prevalence of this disease worldwide and in Iran, we describe a newborn diagnosed with this syndrome at our hospital.

## 2 CASE REPORT

A 26‐year‐old Afghan woman, G3P3L1IUFD1, gestational age: 38^+5^ weeks went to the emergency department complaining of labor pain. She had no underlying disease, and did not mention medication use, with routine pregnancy care. She had a nonconsanguineous marriage. Her first pregnancy was terminated at 24 weeks of gestational weeks due intrauterine fetal demise (IUFD) to severe oligohydramnios and fetal limb and renal anomalies. The parents did not perform a further evaluation to determine the underlying causes of this disorder. The second pregnancy lead to a term female newborn, without any medical problems, weighing 3400 g.

She did not performed aneuploidy screening tests. Ultrasound examination at 13 weeks of gestation manifested a severe oligohydramniosis that there was no evidence of rupture of the membrane in the amniosure test and the vaginal examination. The subsequent sonographic evaluation at 20 weeks of gestation for anomalies showed a single fetus with severe amniotic fluid reduction that caused complete evaluation impossible. The kidneys were echogenic, and the stomach and bladder were not seen. At this time, doctors had recommended pregnancy termination due to poor prognosis, but the parents had declined and did not accept further amniocentesis.

The last two‐dimensional (2D) ultrasound at 32 weeks of gestation showed an IUGR *(Intrauterine growth restriction)* fetus (weight and other parameters less than 2 percent of normal range) with absent umbilical artery diastolic flow. The bladder and stomach were not seen as before. In 38 weeks due to fetal distress shown in NST (nonstress test) as recurrent late deceleration, about 2 hours after admission with cervical dilatation of 2 cm and effacement of 20%, the patient underwent a cesarean section.

**FIGURE 1:**
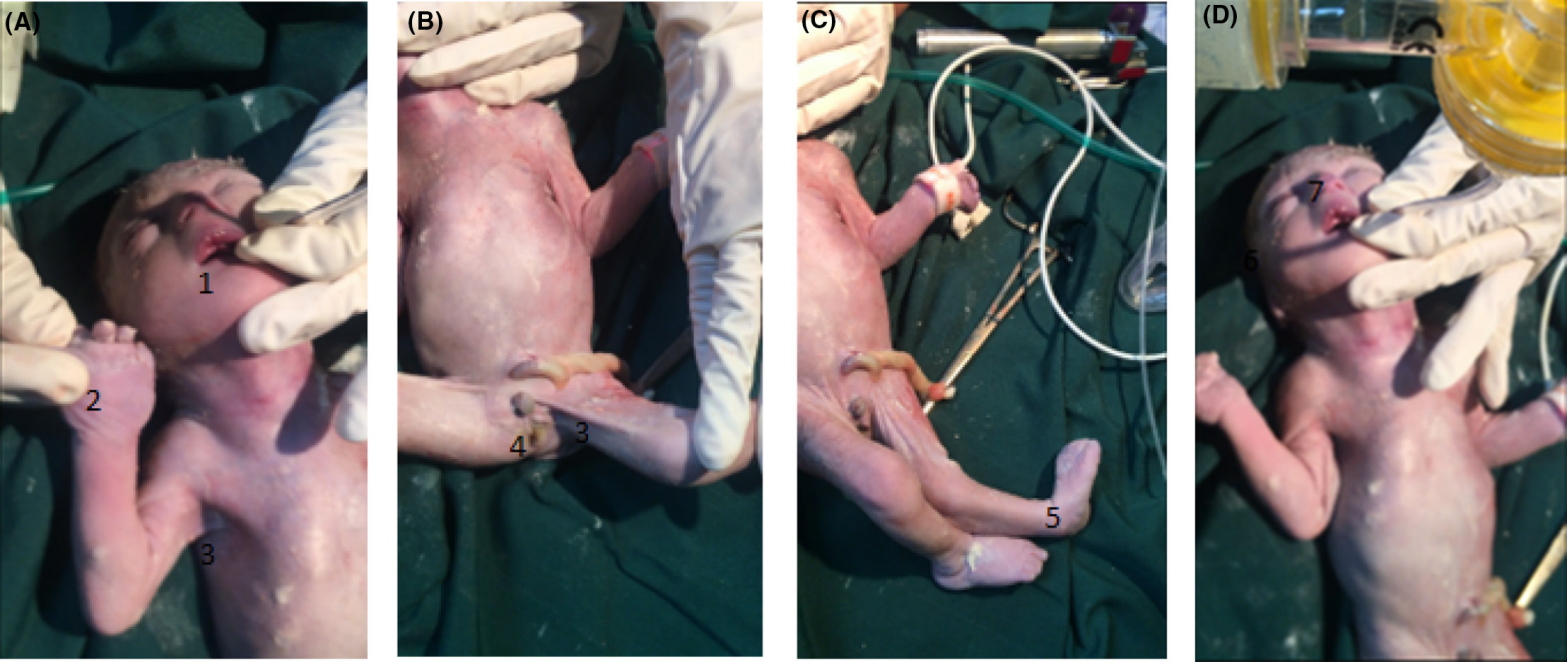
(A–D): Anomalies features that are shown with numbers: 1‐ cleft palate, 2‐ camptodactyly, 3‐ pterygia, 4‐ ambiguous genitalia, 5‐ club foot, 6‐ depressed nasal ridge, and 7‐ low‐set ear.

A dysmorphic, apneic baby boy weighing 900 grams, was born with Apgar 6 in the first minutes and the following anomalies: pterygia involving the axilla, bilateral antecubital fossa, and groin. Arthrogryposis involving multiple lower and upper extremities joints. Cleft palate, microstomia and limitation of mouth opening, webbed neck, asymmetric small and narrow chest, ambiguous genitalia, depressed and wide nasal bridge, antemongoloid slant, low‐set, malformed, and posteriorly rotated ears, pterygia, syndactyly and camptodactyly of hands and rocket bottom feet (Figure 1A–D).

The ABG (atrial blood gas) was 7.25. He required extensive resuscitation at birth, including full cardiopulmonary resuscitation and intubation. After intubation, the chest did not expand properly, and breathing sounds were not heard well. Despite advanced resuscitation, the baby expired about 10 minutes after birth. Whole exome sequencing (WES) showed a homozygous mutation of the CHRNG gene.

## 3 DISCUSSION

LMPS (lethal multiple pterygium syndrome) is characterized by arthrogrypotic contractures of the limbs, pterygia (webbing of the neck, popliteal, and antecubital fossa), lack of muscle movement (akinesia), fetal hydrops with cystic hygroma, short stature, congenital vertical talus, camptodactyly, hip dislocation, facial anomalies, cryptorchidism, pulmonary, cardiac and brain hypoplasia, congenital diaphragmatic hernia, kidney abnormalities, progressive scoliosis, intrauterine growth restriction, and severe oligohydramnios.^1^, ^2^, ^9^

Facial anomalies include depressed nasal bone, hypertelorism, microretrognathism and microstomia, epicanthal folds, low‐set malformed ears, and cleft palate,^4^ cryptorchidism and underdeveloped phallus may be seen in male patients, and females may have missing or underdeveloped labia majora.^5^ In this case, we could observe a large number of these anomalies, as mentioned above.

The main inheritance pattern of the disease is autosomal recessive, which is caused by a mutation in the gamma subunit of the cholinergic receptor (CHRNG) on chromosome 2, as we saw in our case genetic examination. Still, cases of autosomal dominant and X‐linked have also been reported.^6^, ^7^, ^8^ The gamma subunit is expressed in fetal muscles and helps develop neuromuscular junctions. According to studies, it has been determined that there is no relationship between phenotype and genotype, which means that the same mutations can cause both lethal and nonlethal forms.^10^, ^11^, ^12^

The differential diagnosis includes Bartsocas–Papas, FADS (*fetal akinesia deformation sequence*, also known as Pena–Shokeir syndrome type I), Neu–Laxova, and arthrogryposis multiplex; Congenital restrictive myopathy and Bruck syndrome. And the diagnosis is confirmed with genetic examinations.^5^, ^13^

The early diagnosis of MPS is difficult because the only sonographic features present in the first trimester are increased nuchal translucency and fetal hydrops. But we did not find this presentation in second trimester sonography. MPS is often diagnosed in the last months of pregnancy with the manifestations mentioned above, and genetic counseling should be done before the next pregnancy.^14^ LMPS is lethal during pregnancy or soon after birth^4^, ^5^; as we see in our case, that expired about 10 minutes after birth. Knowing how to diagnose and deal with this disorder prevents additional evaluations and investigations and reduces parents' worries. Depending on the inheritance pattern, which is often autosomal recessive, parents should have genetic counseling and can be offered genetic testing and preimplantation genetic tests for future pregnancies.

## 4 CONCLUSION

LMPS is a congenital genetic disease with multiple anomalies that is fatal in the second and third trimesters of pregnancy or shortly after birth. With genetic testing and counseling, it can be prevented from recurring in subsequent pregnancies.

## AUTHOR CONTRIBUTIONS

**Parvaneh Sadeghimoghadam:** Conceptualization. **Saeedeh Shirdel:** Writing – original draft. **Sedigheh Hantoushzadeh:** Supervision. **Zeinab Hashemi:** Writing – review and editing. **Marjan Ghaemi:** Conceptualization.

## FUNDING INFORMATION

None.

## CONFLICT OF INTEREST STATEMENT

The authors have no conflict of interest to declare.

## CONSENT FOR PUBLICATION

Written informed consent was obtained from the patient to publish this report in accordance with the journal's patient consent policy.

## Data Availability

Data is available upon request.
